# Keratoelastoidosis Marginalis of the Hands: A Distinct Form of Palmoplantar Keratoderma

**DOI:** 10.7759/cureus.73515

**Published:** 2024-11-12

**Authors:** Andreia Coutada, Mirjam R Butina, Bostjan Luzar

**Affiliations:** 1 Pathology, Portuguese Oncology Institute of Porto, Porto Comprehensive Cancer Centre Raquel Seruca, Porto, PRT; 2 Dermatology, Dermatologija Rogl Fabjan, Ljubljana, SVN; 3 Dermatopathology, Institute of Pathology, Faculty of Medicine, University of Ljubljana, Ljubljana, SVN

**Keywords:** clinical dermatology, collagenous and elastotic plaques, hand dermatosis, keratoelastoidosis marginalis, keratosis

## Abstract

Keratoelastoidosis marginalis of the hands (KEMH) is an acquired form of marginal papular keratoderma, characterized by thickened keratotic plaques predominantly affecting the lateral side of the index finger and the medial side of the thumb. It is often associated with chronic sun exposure and trauma, usually affecting older individuals. Due to clinical similarities with other palmoplantar keratodermas, differential diagnosis is essential for effective treatment management. Although clinical information is often sufficient for differentiation, a skin biopsy can provide valuable diagnostic clues. We report the case of a 63-year-old male patient who presented with rough linear hyperkeratotic lesions on the lateral and medial margins involving the first and second fingers of both hands, which had developed over a period of three years. Skin biopsy revealed orthohyperkeratosis with an underlying epidermis of normal thickness, with no features of actinic keratosis. In the dermis, thickened elastic fibers and degenerated collagen bundles were haphazardly distributed. Based on clinicopathological findings, a diagnosis of KEMH was made. As limited information is available in the literature, we aim to expand the current understanding of KEMH by emphasizing crucial aspects of its pathogenesis, histological characteristics, and main differential diagnoses.

## Introduction

Keratoelastoidosis marginalis of the hands (KEMH) is a rare acquired dermatological condition that belongs to the category of marginal papular keratodermas (MPK), from the vast group of palmoplantar keratodermas (PPK) [1]. KEMH was initially reported as “degenerative collagenous plaques of the hands” by Burks et al. [2] in 1960, who described distinct symmetrical, bilateral, linear, and confluent plaques and/or papules of the index fingers. On histology, Burks et al. observed a thickened epidermis with hyperkeratosis and the presence of dense collagen bundles mixed with elastic fibers in the reticular dermis, which they attributed to senile degeneration of the sun-exposed skin. Later in 1965, Kocsard [3] introduced the term “keratoelastoidosis marginalis of the hands” for the same condition and concluded that chronic pressure from manual labor was the main etiological factor leading to the formation of these plaques. Afterward, Mehregan [4] proposed that a combination of long-term trauma and pressure, along with some degree of actinic exposure, was responsible for the appearance of these marginal palmar lesions, which are now generally accepted as the main etiologic factors [5,6]. Additional terms used in the literature include “digital papular calcific elastosis” (DPCE) [7] and “collagenous and elastotic marginal plaques of the hands” [6].

## Case presentation

**Figure 1:**
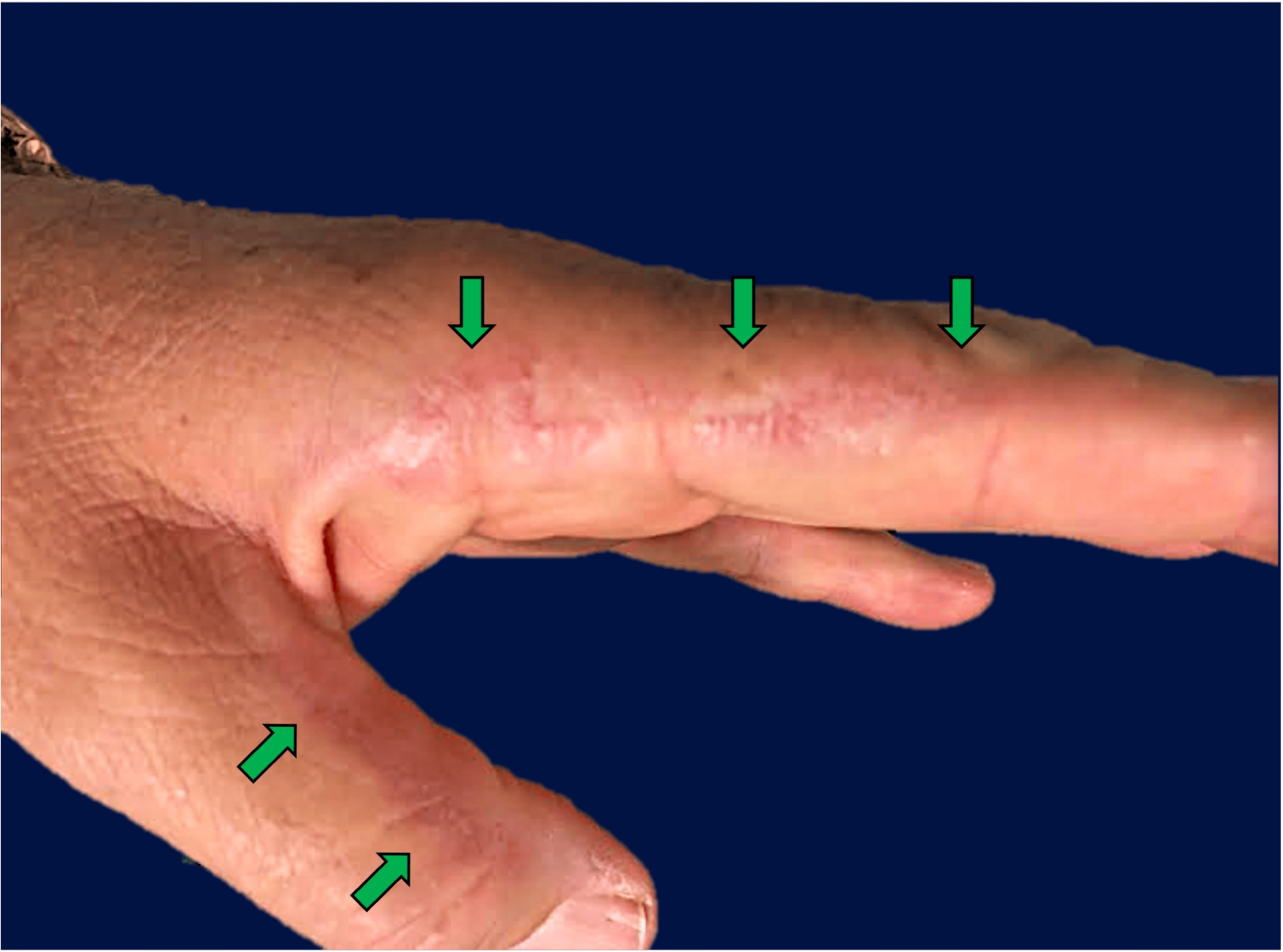
Gross characteristics of keratoelastoidosis marginalis of the hands Physical examination reveals rough linear hyperkeratotic lesions at the junction of the dorsal and palmar skin on the radial side of both hands, extending distally from the medial aspect of the thumb in continuity to the radial aspect of the index finger (arrows).

A 63-year-old male patient presented to a dermatology clinic for rough linear hyperkeratotic lesions on the lateral and medial margins involving the first and second fingers of both hands (Figure 1), which had developed over a period of three years. The patient attributed their occurrence to frequent hand washing and disinfection during the COVID-19 pandemic. Over the following years, these lesions were treated as an uncommon detritive dermatitis/dyshidrosis with topical corticosteroids and keratolytics, without significant results. The patient is a retired merchant and has denied contact with irritants/allergens, trauma, or frequent sun exposure, at least in the last decades. However, in adolescence, he frequently played football and tennis outdoors whenever possible. The patient has been treated with nilotinib for chronic myeloid leukemia, diagnosed 10 years ago, and is regularly taking allopurinol, antilipemic, and antihypertensive drugs.

General physical examination, besides hyperkeratotic lesions on both hands described, showed signs of intrinsic aging with a moderate amount of solar lentigines and seborrheic keratoses on the face and body. Blood and urine exams were both normal.

**Figure 2:**
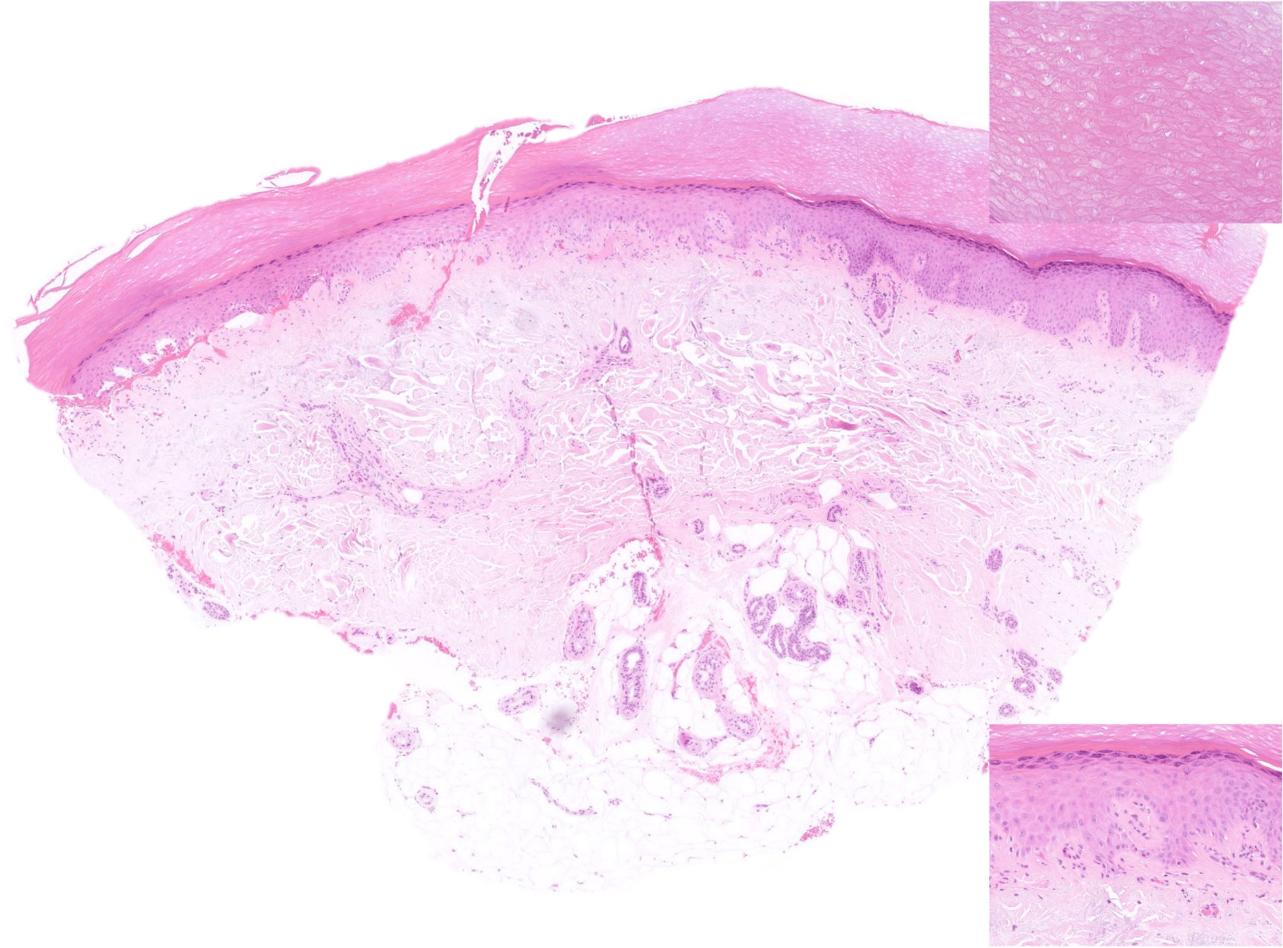
Histological characteristics of keratoelastoidosis marginalis of the hands Skin biopsy from the keratotic plaques on the fingers shows compact orthohyperkeratosis (upper inset) and normal thickness of the epidermis, with no features of actinic keratosis (bottom inset). The subcutaneous tissue shows no histological changes.

**Figure 3:**
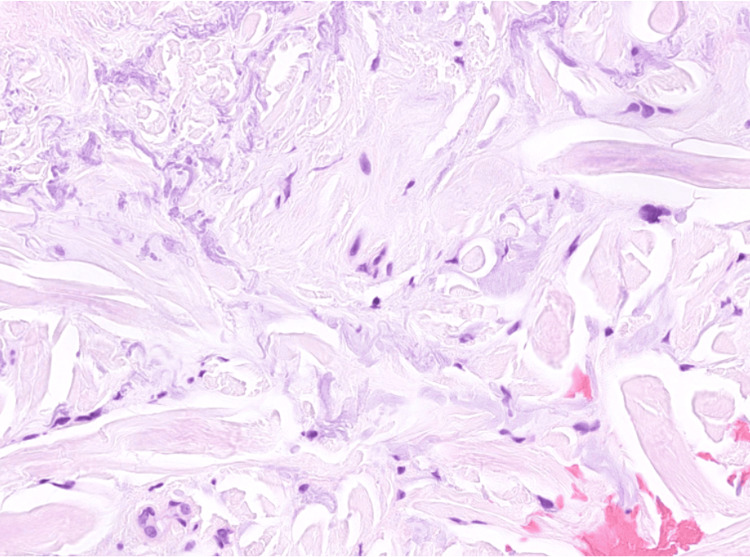
Histological characteristics of keratoelastoidosis marginalis of the hands Fragments of thickened elastic fibers are haphazardly distributed throughout the dermis. No calcifications or lymphocytic infiltration are observed.

**Figure 4:**
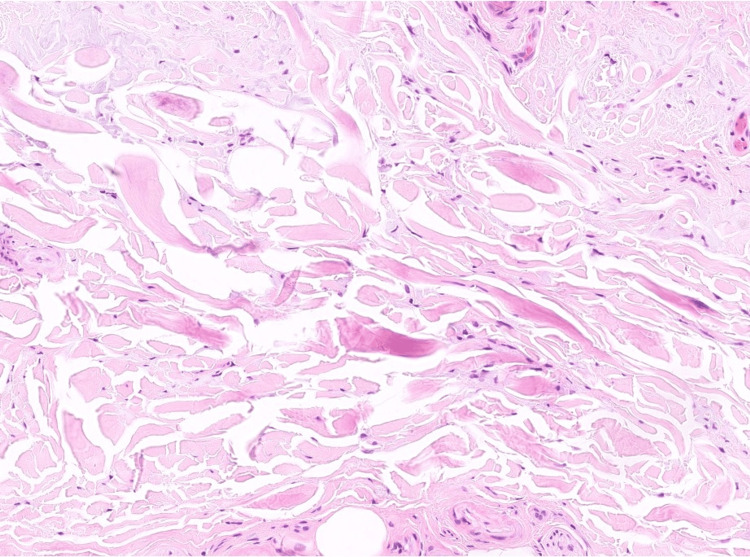
Histological characteristics of keratoelastoidosis marginalis of the hands Fragments of thickened and degenerated collagen bundles surrounded by retraction artifacts, haphazardly distributed throughout the dermis. No calcifications or lymphocytic infiltration are observed.

**Figure 5:**
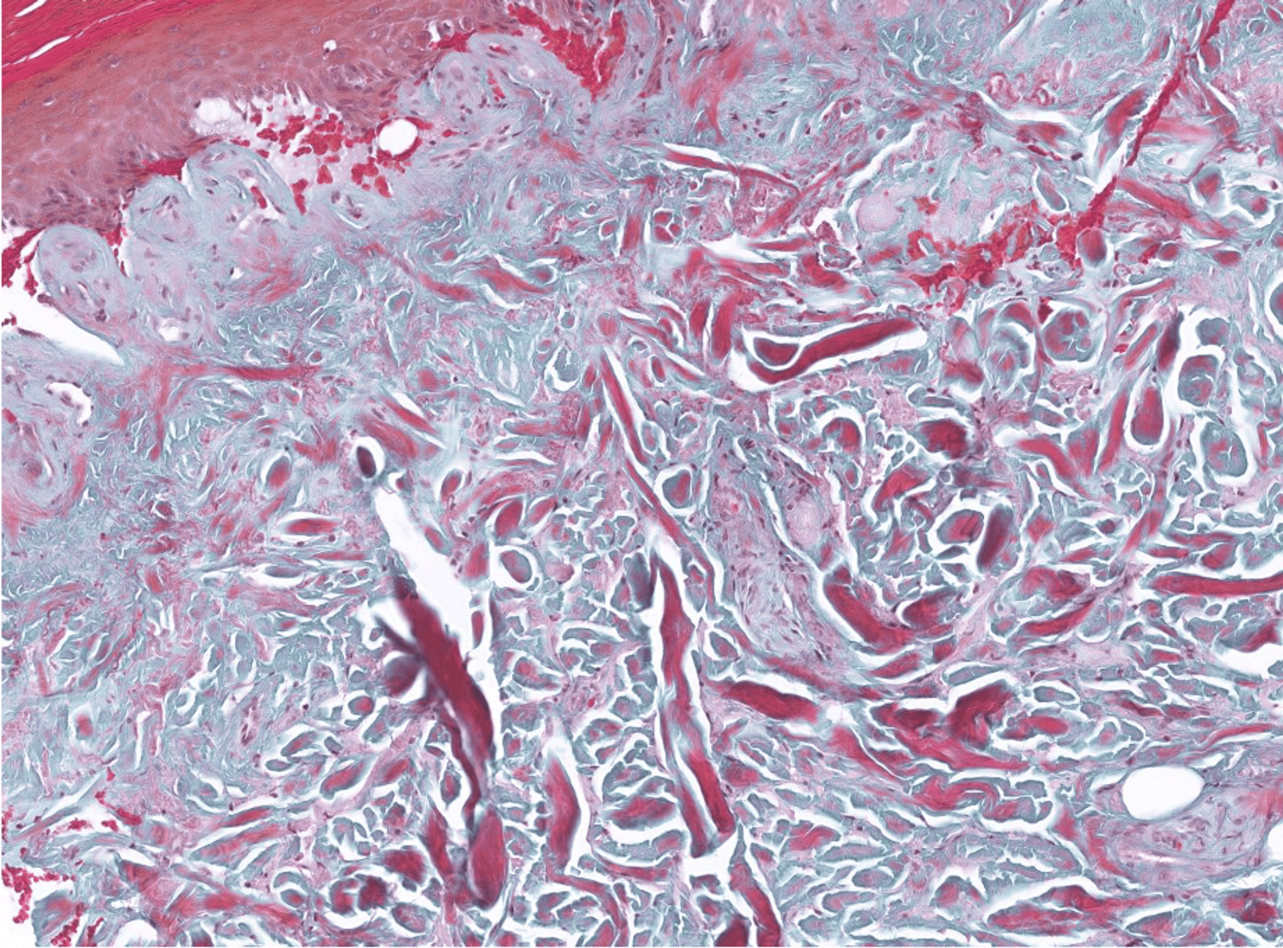
Goldner's trichrome stain Goldner's trichrome stain reveals thick collagen bundles irregularly dispersed in the dermis, occasionally aligning perpendicular to the epidermis.

**Figure 6:**
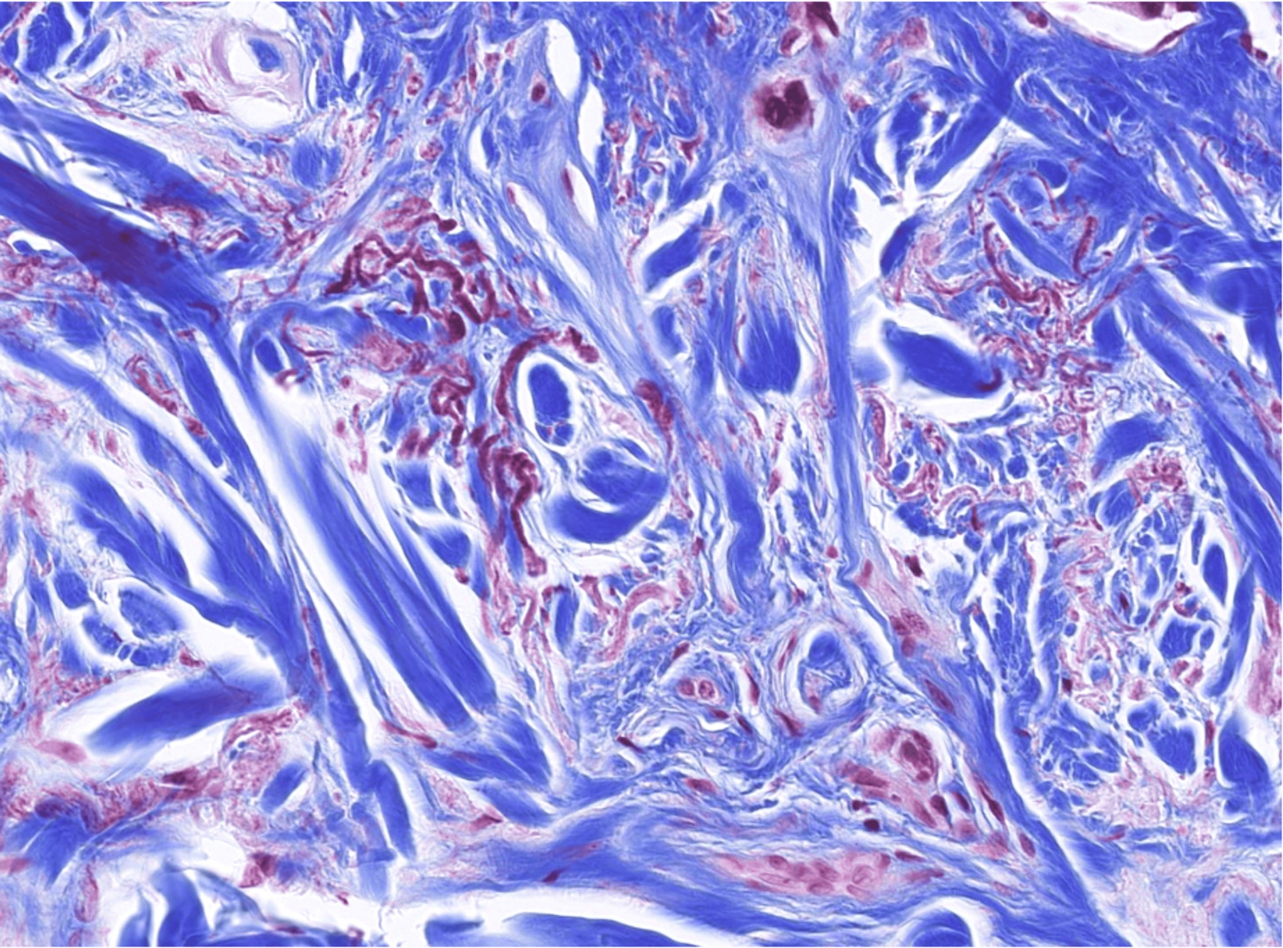
Masson's trichrome stain Masson's trichrome stain reveals thick collagen bundles irregularly dispersed in the dermis.

**Figure 7:**
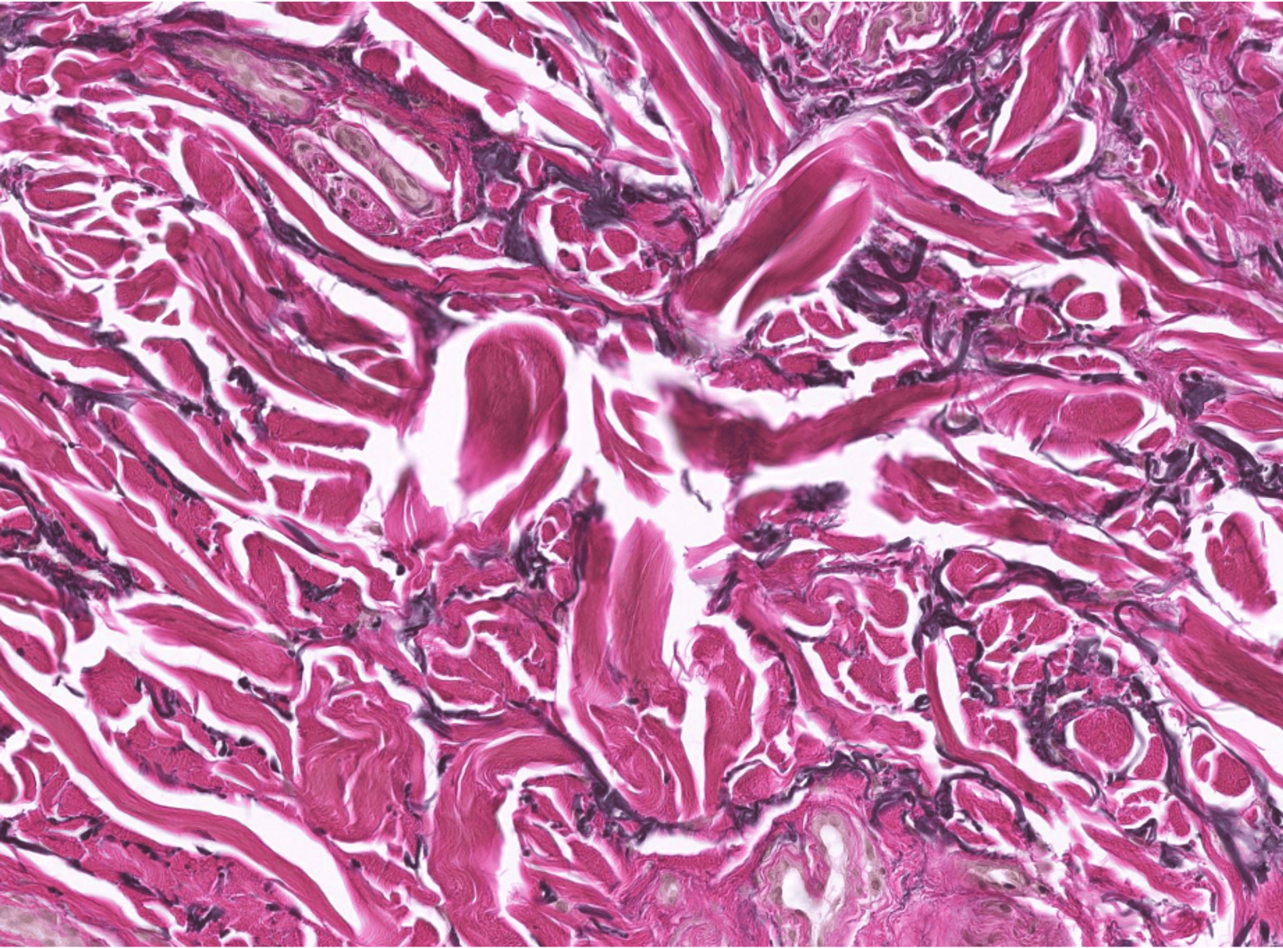
Verhoeff-van Gieson stain Verhoeff-van Gieson stain highlights the fragmentation of elastic fibers.

Histological examination of the skin biopsy showed compact orthohyperkeratosis and normal thickness of the epidermis (Figure 2), with no features of actinic keratosis. In the papillary and reticular dermis, fragments of thickened elastic fibers and degenerated collagen bundles were haphazardly distributed throughout the dermis (Figures 3-4), without accompanying lymphocytic infiltration. Special stains using Goldner's trichrome (Figure 5) and Masson's trichrome (Figure 6) revealed thick collagen bundles irregularly dispersed in the dermis, occasionally aligning perpendicular to the epidermis, while the Verhoeff-van Gieson (Figure 7) stain highlighted the fragmentation of elastic fibers. Based on clinicopathological findings, a diagnosis of KEMH was made.

## Discussion

PPKs represent a heterogenous group of hereditary and acquired disorders of cornification, characterized by prominent hyperkeratosis of the skin on the palms and soles [1]. Based on distinct patterns of palmoplantar involvement, PPKs can be classified into diffuse, focal, striate, and punctate variants [1,8].

KEMH is a form of MPK that is part of the group of punctate PPKs [1], characterized by multiple papular lesions with epidermal thickening, scattered or aggregated, mostly in a linear distribution along the edges of the hands and feet. These lesions may also occasionally extend to the dorsal areas [1,8]. Given the multitude of entities that frequently share keratotic papules along the border of hands and feet, Rongioletti et al. [9] proposed a simplified classification for MPKs in 1994, dividing them into hereditary and acquired types. The hereditary type was further subdivided based on the presence or absence of elastorrhexis, a condition characterized by fragmentation and diminished density of elastic fibers in the skin. Elastorrhexis is a distinct feature that characterizes acrokeratoelastoidosis [10], distinguishing it from the rest of hereditary MPKs, such as focal acral hyperkeratosis (FAH), hereditary papulotranslucent keratoderma, acrokeratoderma hereditarium punctatum, and mosaic acral keratosis [9]. Because of the clinical similarities among hereditary MPKs, Abulafia and Vignale [11] advocated that these conditions may reflect variable genetic expressions of the same underlying condition and, therefore, should be considered variants rather than separate entities. KEMH, along with DPCE, is part of the acquired type of MPKs proposed by Rongioletti et al. [9], though the authors suggested that DPCE may be a variant of KEMH with additional features of calcium deposition in degenerated elastic fibers [7].

The pathogenesis of KEHM has not been completely elucidated. Initially, Burks et al. [2] regarded KEMH as a degenerative change of unknown etiology, most likely but not exclusively linked to actinic degeneration. Later, it was suggested that KEMH was merely a form of solar elastosis [12], a theory that was refuted by other authors [3,7,13], who proposed that KEMH is a unique degenerative disease involving both collagen and elastotic components.

KEMH usually affects middle-aged and elderly individuals [3], with males being more commonly affected than females in the white population [14]. Chronic sun exposure and long-term trauma or pressure, primarily due to manual labor, appear to be the main contributing factors [4-6], even though they have not been universally reported among all affected patients with KEMH [6,11]. In our patient's case, the history of chronic sun exposure or repetitive trauma was somehow established. Despite these factors being commonly linked to KEMH, their absence doesn't rule out the diagnosis. Other circumstances, like hypothyroidism and the use of targeted therapies for the treatment of some malignancies, including tyrosine kinase inhibitors, have been linked with the development of PPKs [1]. With the fact that our patient is taking nilotinib, one could consider whether this medication could have influenced the development or exacerbation of his condition; however, there is no reported data in the literature to support this association. Therefore, while exploring potential causes, it is important to consider other factors beyond sun exposure and trauma, such as genetic predisposition or the effect of certain drugs.

Clinically, KEMH presents as asymptomatic, bilateral, and symmetrical linear white to yellowish papules and/or plaques at the junction of the dorsal and palmar skin on the radial side of the hand, extending distally from the medial aspect of the thumb in continuity to the radial aspect of the index finger [2-7,11-15]. Recently, KEMH has also been described on the feet [16], yet this presentation is rare.

Histological findings of KEMH may include acanthosis of the epidermis with overlying orthohyperkeratosis, which can result from repeated episodes of hypoxia resulting from papillary dermal capillary occlusion during periods of pressure, combined with compression from elastotic material that accumulates in the dermis [3]. In the dermis, there are thick, haphazardly arranged collagen bundles, sometimes perpendicular to the epidermis, often surrounded by retraction artifacts and elastic fragmentation [2-7,11-15], as is also evident in our case. Additionally, some authors described the presence of basophilic elastotic masses that are often calcified [7], a feature not encountered in our case. An inflammatory infiltrate can occasionally be present in the dermis [14].

One of the most common and somewhat confusing differential diagnoses for KEMH is the acrokeratoelastoidosis of Costa [17], first described in 1952. Clinical history and the age of onset are more important than histology in differentiating these two pathogenically different entities. Acrokeratoleastoidosis typically occurs in younger patients and can affect the lower extremities, whereas KEMH is classically seen in older patients and predominantly affects the hands [1,5,11]. Acrokeratoelastoidosis occasionally extends to the dorsal aspect of hands and feet, a feature not commonly seen in KEMH [1,6,8]. Histologically, acrokeratoelastoidosis shows a loss of elastic tissue, in contrast to KEMH, which demonstrates an increase [11,13]. Other differential diagnoses include FAH, which also affects both hands and feet, but on histology, changes are limited to the epidermis and do not involve the dermis [5,9]. Pseudoxanthoma elasticum can also show calcification of elastic fibers similar to KEMH, but occurs in flexural folds and does not affect the hands [5].

For most of the patients, KEMH is merely a cosmetic problem. Primary prevention of actinic-related damage is one of the most effective strategies for the management of KEMH [5]. Treatment options include laser therapy, topical keratolytics or retinoids, oral isotretinoin, and cryotherapy, however, with variable success [14].

## Conclusions

This rare case of KEMH highlights the importance of accurate differential diagnosis due to its clinical similarities with other subtypes of PPKs in order to warrant targeted and effective treatment management. Although clinical information is often sufficient for differentiation, a skin biopsy can provide valuable diagnostic clues. While chronic sun exposure and trauma are commonly recognized as the main causes, this case also suggests the need to consider other factors like drug effects, particularly in patients with a history of targeted cancer therapy.
